# Household

**Published:** 1887-02

**Authors:** 


### HOUSEHOLD.

Coffee Rolls.—Take one quart of bread dough and mix with it one-half cup of sugar, one-quarter cup of butter, two cups of dried currants; make into small rolls, dip into melted butter, place in tins and let rise and bake.

Broiled Oysters.—Dry the oysters in a napkin, season with pepper and salt, and broil on a wire-folding grid-iron, turning frequently. Serve immediately in a hot dish, with butter.

Curried Oysters.—Put the liquor drained from the oysters in a sauce-pan, with half a cup of butter, two tablespoonfuls of flour, and one of curry powder; let boil; add the oysters and serve at will.

Tomato Soup.—To one pint of canned tomatoes, or four large ones cut up fine, add one quart of boiling water, and let them boil till done; then add nearly a teaspoonful of soda; when it foams up add one pint of sweet milk, pepper and butter, or one cup of sweet cream instead of butter, a few crackers rolled fine, and serve.

Breaking Glass Any Required Shape.—Make a small notch by means of a file on the edge of a piece of glass, then make the end of a tobacco pipe, or of a rod of iron of the same size, red hot in the fire; apply the hot iron to the notch and draw it slowly along the surface of the glass in any direction you please—a crack will follow the direction of the iron.

To Keep Lemons.—Lemons are a very cheap luxury for those living near cities, or having easy access to rapid transportation, and can be kept fresh for months by putting them into a clean tight jar or cask, and covering them with cold water. Keep in a cool place out of reach of sunlight, and change the water often, not less than every third day; every second day is better. Lemons are excellent for winter use, or if one is bilious or inclined to rheumatism.

Ivory may be cleaned by scrubbing with a new soft tooth-brush, soap and tepid water, then dry the ivory and brush well, dip the latter in alcohol and polish the ivory until it has regained its former sheen. If the water gives the ivory a yellowish tint dry the object in a heated place. If age has yellowed it, place the object under a bell-jar with a small vessel containing lime and muriatic acid, and set the whole in the sunshine.

Apple Pudding.—Cut good, tart cooking apples into slices, after they are peeled and cored, and lay them in a buttered baking dish, in alternate layers with dry bread crumbs. Sprinkle each layer thickly with sugar and lightly with cinnamon, and let the top layer be bread crumbs. Melt an ounce of butter and pour over the pudding. Bake till the apples are done. This recipe may be varied by using apples for the top layer, and covering the pudding, just after taking from the oven, with a meringue made by beating the whites of three eggs to a froth, with two tablespoonfuls of granulated sugar and the juice of half a lemon. Return it to the oven long enough for the eggs to acquire the desired firmness.

How to Press Embroidery—Ordinary flat embroidery may be pressed with a hot iron on the wrong side, laying the piece on a damp cloth; but as this treatment would ruin raised work, like ribbon embroidery, arrasene work, etc., a better way is to lay a wet towel on the table or the carpet; spread over this the piece of work right side up, and tack tightly to the floor, taking care to draw it tight enough to remove all wrinkles, let it dry in this position. Some draw work of this kind into shape by holding it over boiling water and steaming it, and then tack out on the carpet as described.

Restoring Plush.—It is customary to use ammonia for the purpose of neutralizing acids that have accidently or otherwise destroyed the color of fabrics. This must be applied immediately or the color is usually imperfectly restored. After careful use an application of chloroform will bring out the colors as bright as ever. Plush goods and all articles died with analine colors, faded from exposure to light, will look as bright as ever after sponging with chloroform. The commercial chloroform will answer the purpose just as well. This chloroform will be found very useful, as chloroform, which is quite cheap, readily restores the color of faded plush garments that have been consigned.

Apple Meringue.—Prepare six large, tart apples for sauce. While hot put in a piece of butter the size of an egg. When cold, add a cup of fine cracker crumbs, the yolks of three eggs well beaten, a cup of sweet milk or cream, a little salt, nutmeg and sugar to taste. Bake in a large plate, with an undercrust of rich paste and a rim of puff paste. When done, take the whites of the eggs, half a teacup of white sugar and a few drops of essence of lemon; beat to a stiff froth, pour over and put back into the oven to brown lightly.

To Clean Kid Gloves.—Stains may be removed from even the most delicately colored kid gloves, without injury, by suspending them for a day in an atmosphere of ammonia. Provide a tall glass cylinder, in the bottom of which place strong aqua ammonia. Be careful to remove from the sides of the jars any ammonia that may be spattered upon them. Suspend the gloves to the stopper in the jar. They must not come in contact with the liquid.

Mince meat is now put up dry, in sealed packages. It is said to be of superior quality, and will keep any length of time, winter or summer, in any climate, and especially and peculiarly adapted for spring and summer use; no risk of spoiling in warm weather; always ready and handy for use.

To remove mildew, rub common yellow soap on the damaged article, and then salt and starch on that. Rub well and put out in the sunshine.

